# Effect of supplementary zinc on orthodontic tooth movement in a rat model

**DOI:** 10.1590/2177-6709.21.2.045-050.oar

**Published:** 2016

**Authors:** Ahmad Akhoundi Mohammad Sadegh, Ghazanfari Rezvaneh, Etemad-Moghadam Shahroo, Alaeddini Mojgan, Khorshidian Azam, Rabbani Shahram, Shamshiri Ahmad Reza, Momeni Nafiseh

**Affiliations:** 1 Professor, Tehran University of Medical Sciences, Dental Research Center, Orthodontic Department, Tehran, Iran.; 2 Postgraduate student, Tehran University of Medical Sciences, Prosthodontic Department, Tehran, Iran.; 3 Associate Professor, Tehran University of Medical Sciences, Dental Research Center, Dentistry Research Institute, Tehran, Iran.; 4 Dentist, Tehran University of Medical Sciences, Dental Research center, Dentistry Research Institute, Tehran, Iran.; 5 Head of Experimental Research Laboratory, Tehran University of Medical Sciences, Tehran Heart Center, Tehran, Iran.; 6 Tehran University of Medical Sciences, Research Center for Caries Prevention, Dentistry Research Institute, Department of Community Oral Health, Tehran, Iran.

**Keywords:** Dietary supplement, Orthodontics, Tooth movement, Zinc

## Abstract

**Introduction:**

Osteoclasts and osteoblasts are responsible for regulating bone homeostasis during which the trace element zinc has been shown to exert a cumulative effect on bone mass by stimulating osteoblastic bone formation and inhibiting osteoclastic bone resorption.

**Objective:**

The aim of the present study was to investigate the effects of zinc (Zn) on orthodontic tooth movement (OTM) in a rat model.

**Material and Methods:**

A total of 44 male Wistar rats were divided into four groups of 11 animals each and received 0, 1.5, 20 and 50 ppm Zn in distilled water for 60 days. In the last 21 days of the study, nickel-titanium closed coil springs were ligated between maxillary right incisors and first molars of all rats, and tooth movement was measured at the end of this period. Histological analysis of hematoxylin/eosin slides was performed to assess root resorption lacunae, osteoclast number and periodontal ligament (PDL) width.

**Results:**

Mean OTM was calculated as 51.8, 49.1, 35.5 and 45 µm in the 0, 1.5, 20 and 50 ppm zinc-receiving groups, respectively. There were no significant differences in neither OTM nor histological parameters among the study groups (*p* > 0.05).

**Conclusion:**

According to the results obtained in the current investigation, increase in supplementary zinc up to 50 ppm does not affect the rate of OTM neither bone and root resorption in rats.

## RESUMO

**Introdução:**

os osteoclastos e os osteoblastos são responsáveis por regular a homeostase óssea, processo no qual o oligoelemento zinco tem demonstrado exercer um efeito cumulativo sobre a massa óssea, estimulando a formação óssea osteoblástica e inibindo a reabsorção óssea osteoclástica.

**Objetivo:**

o objetivo do presente estudo foi investigar os efeitos do zinco (Zn) sobre a movimentação dentária ortodôntica (MDO) em ratos.

**Métodos:**

um total de 44 ratos Wistar machos foi dividido em quatro grupos de 11 animais cada, os quais receberam 0; 1,5; 20 e 50ppm de zinco diluído em água destilada, durante 60 dias. Nos últimos 21 dias do estudo, molas helicoidais fechadas de níquel-titânio foram instaladas entre os incisivos direitos e os primeiros molares superiores de todos os ratos, e a movimentação dentária foi medida ao final desse período. Foi realizada análise histológica de cortes corados por hematoxilina-eosina, para avaliar as lacunas de reabsorção radicular, o número de osteoclastos e a espessura do ligamento periodontal.

**Resultados:**

as médias da MDO foram estimadas em 51,8; 49,1; 35,5 e 45µm no grupos que receberam, respectivamente, 0; 1,5; 20 e 50ppm de zinco. Não houve diferença significativa entre os grupos experimentais, nem quanto à MDO, nem quanto aos parâmetros histológicos (*p* > 0,05).

**Conclusão:**

segundo os resultados obtidos na presente investigação, verificou-se que um aumento na dose de suplementação com zinco para 50ppm não afeta nem o índice de MDO, nem a reabsorção óssea ou radicular em ratos.

## INTRODUCTION

Zinc (Zn) is an essential trace element that serves as a cofactor for more than 200 enzymes, and being a constituent of nearly all human cell types, plays a major role in a number of basic biological processes including proliferation, wound healing, immunity and osteogenesis.^1^ Deficiency of this fundamental mineral is a universal health issue, especially during adolescence due to the occurrence of growth spurts. This has led to the recognition of a need for improved public health programs to support individuals with Zn deficiency known to comprise half the world's population.^2^ Delayed bone maturation and impaired growth are two of the major consequences of insufficient Zn intake in pubescent individuals who are being treated worldwide by prescription of Zn supplements as part of their treatment regimen.^3^ In addition to the bone-related applications of this substance, different compounds, such as zinc gluconate glycine and zinc acetate, are routinely used as anti-cold agents.^4^

Zn impacts bone metabolism via augmentation of osteoblastic activity and down regulation of osteoclastic bone resorption, a fact reported by numerous investigations.^5^ ^-^ ^8^ Bone remodeling is the foundation of orthodontic treatment and tooth movement relies on this phenomenon.^9^ Following the application of orthodontic forces, the periodontium responds by an inflammatory reaction leading to reorganization of its cellular components and a modification in its equilibrium in favor of bone remodeling, the end result of which would be tooth movement.^10^ ^,^ ^11^ ^,^ ^12^

Several authors have studied the effects of local and systemic medicaments, including dietary supplements on orthodontic tooth movement (OTM).^13^ ^-^ ^16^ A considerable number of patients seeking orthodontic treatment may be using medications due to general health problems; moreover, regarding the prevailing trend towards the increased use of dietary supplements among these individuals, having some notion of the effect of various drugs on OTM would be helpful for treatment planning and predicting the length of these treatment modalities.^17^ Considering the osteogenic potential of Zn, along with its inhibiting impact on bone resorption,^7^ it was hypothesized that this substance might negatively regulate the rate of OTM in rats. Since Zn supplementation is becoming prevalent among patients, the present study was designed to investigate the effect of this micronutrient on tooth movement in rats.

## MATERIAL AND METHODS

### Animals

The experimental protocol of the current study was approved by the Ethics Committee of Tehran University of Medical Sciences (code: 91-01-70-17586-55909). A total of 44 male Wistar rats (200-250 g) were housed in plastic cages, maintained on a 12/12 hour light-dark cycle and randomly divided into four groups (n = 11) with free access to standard laboratory chow. Their drinking water consisted of double distilled water with Zn sulfate added at concentrations of 0, 1.5, 20 and 50 ppm for use in the control group and groups 1, 2 and 3, respectively.^18^ ^,^ ^19^ ^,^ ^20^ All animals were weighed at the beginning of the study (day 1), on the first day of appliance placement (day 40) and immediately before sacrifice (day 60). 

### Orthodontic treatment and measurement of tooth movement

On day 40^th^ of the study period, each rat was anaesthetized with an intraperitoneal injection of xylazine HCL (6 mg/kg body weight) and ketamine (50 mg/kg body weight) in order to receive orthodontic appliances. Based on the method suggested by Nilforoushan et al,^22^ nickel-titanium (NiTi) closed coil springs (NiTi, 3M Unitek, Monrovia, CA, Hitek, 0.006 × 0.022-in) were ligated between left maxillary first molars and incisors of all rats with 0.010-in stainless steel ligature wires to deliver a force of 60 g without further activation throughout the duration of the investigation. Labial and distal grooves cut, in approximation to the gingival margins of incisors, were used to retain the wires. The mesiolingual undercut of the first molar provided necessary retention in the posterior segment of the appliance. Two incisors were attached together by means of composite resin (Transbond XT, 3M Unitek, Monrovia, Calif) to achieve anterior anchorage and ensure mesial movement of molars.^21^ Moreover, composite resin covered the anterior ligatures to preserve the wires during the study period. This was followed by 1.5 mm reduction of mandibular incisors with a high-speed handpiece in order to prevent severance of the ligature wires.^22^ ^,^ ^23^ At the beginning of the study, none of the animals demonstrated any kind of space between first and second molars and all contacts were intact.

After orthodontic treatment, the standard rat chow was ground to provide a soft diet for the purpose of minimizing any discomfort and diminishing the chance of dislodgement or damage to the appliances.

All animals were sacrificed on day 60^th^ of the study period by ether overdose followed by decapitation. A feeler gauge was employed to assess mesial movement of first molar by measuring the space between first and second molars before removal of the appliances, so as to prevent any possible distal relapse of the first molar. All measurements were repeated twice by the same operator blinded to the study groups, and the means were used for statistical analysis.

### Histological evaluation

The maxillae were separated, fixed in 10% formalin for five days and immersed in 5% formic acid until adequately decalcified (an average of five days). Sequential 5-µm serial sections were prepared from each paraffin block and the five sections containing the largest root area were chosen and analyzed histomorphometrically, as described previously.^24^ ^,^ ^25^ The final value was expressed as the mean of the selected sections.^26^ The mesial root was histomorphometrically evaluated on the section containing the full length of the root from the cemento-enamel junction (CEJ) to the apex, by means of a double-headed Olympus BX-41 light microscope equipped with a digital camera (DP25 Olympus) and analysis software (DP2-BSW, Olympus). The number of osteoclasts, periodontal ligament (PDL) width, number of resorption lacunae and their depths and widths were analyzed by two observers, and disagreements were solved by consensus.^27^ The width of the PDL was determined coronally and apically on both mesial and distal aspects of the mesial root.^27^ ^,^ ^28^ All sections were measured twice by both observers on the double-headed microscope, and the mean of the two values was used in all consecutive calculations.

### Statistical analysis

Differences among groups were analyzed by one-way ANOVA followed by Tukey post-hoc tests for multiple comparisons. Probability values *p* < 0.05 were considered statistically significant.

## RESULTS

There was a gradual increase in rats' weight during our investigation, and none of them died or demonstrated weight loss throughout the study period. No significant differences in mean overall weights were found among the four study groups (*p* = 0.25). All treated first molars shifted mesially into the space between molars and incisors (Table 1). The highest and lowest amounts of tooth movement were observed in the control and 20 ppm Zn groups, respectively; but there were no significant differences among groups (*p* = 0.18).

**Table 1:** Molar separation over 21 days.

Group	n	Molar separation			95% CI		Minimum	Maximum
		Mean (µm)	SD	SE	Lower	Upper		
50 ppm zinc*	11	450	202	67	295	605	150	650
20 ppm zinc	11	355	149	45	254	455	150	600
1.5 ppm zinc	11	491	189	57	364	618	150	700
0 ppm zinc	11	518	186	56	393	643	250	850

*Zinc was added to distilled water.

Descriptive histological data are shown in Table 2. Osteoclast numbers and resorptive lacunae widths and depths showed no significant differences among study groups (Table 2). Of the four measured locations of PDL width, significant difference was found only in the disto-apical area (Table 2) among groups. Based on multiple comparisons, PDL width was significantly higher in group 1 (1.5 ppm) compared to the control group (*p* = 0.02). 

**Table 2:** Descriptive histological data.

	Osteoclasts number Mean (SD)		Number of RL* Mean (SD)		RL width Mean (SD) (µm)		RL depth Mean (SD) (µm)	
	Mesial	Distal	Mesial	Distal	Mesial	Distal	Mesial	Distal
50 ppm zinc	1.5 (1.9)	1.2 (2.2)	0.8 (0.7)	0.8 (1.2)	57.7 (84.0)	69.1 (105.8)	31.4 (49.1)	26.0 (36.7)
20 ppm zinc	1.5 (1.7)	1.6 (1.6)	0.5 (0.7)	0.7 (0.8)	60.4 (66.5)	70.9 (77.2)	33.2 (41.4)	27.3 (27.9)
1.5 ppm zinc	0.9 (1.6)	1.2 (1.7)	0.9 (1.0)	0.8 (0.8)	88.5 (98.1)	41.7 (41.6)	24.0 (30.5)	28.0 (29.3)
0 ppm zinc	0.3 (0.6)	0.0 (0)	1.0 (1.5)	1.0 (1.5)	45.2 (74.9)	75.4 (110.9)	17.3 (26.9)	21.0 (28.3)
*p* value	0.14	0.10	0.58	0.97	0.67	0.82	0.75	0.95

* RL = Resorption lacuna.

## DISCUSSION

Zn supplements are prescribed for adults for numerous reasons. Among the various nutritional attributes of Zn, inhibition of bone resorption and stimulation of bone growth and mineralization^7^ might directly influence OTM induced by orthodontic treatment. Several studies have investigated the effects of Zn on various aspects of bone quality,^5^ ^,^ ^29^ and its deficiency has been suggested to play a role in the development of osteoporosis.^30^

In the present study, we measured the amount of tooth movement in rats receiving 0 to 50 ppm zinc sulfate, followed by application of a simple orthodontic appliance, and did not observe significant difference among groups, which was also confirmed by our histomorphometric analysis. The present result was in agreement with a study conducted by Abrisham et al^8^ who also did not find Zn to be effective in bone healing and reported no significant relationship between this substance and bone formation in rabbits. Similarly, a periodontal study in rats failed to demonstrate differences in pocket depths between animals receiving Zn-containing diets and those deficient in this micronutrient.^31^ In contrast to our findings, Zn has been shown to prevent osteoporosis^32^ and induce osteogenesis.^5^

Previous investigations have indicated that the duration of Zn application may have an impact on its expected bone effects. Accordingly, any possible positive function of this micronutrient diminishes with time.^8^ ^,^ ^33^ This may explain the lack of difference in OTM between control and Zn-receiving rats; consequently, the 40^th^ day period of Zn administration prior to orthodontic treatment in the current investigation may have suppressed the activity of this element. For the same reason, it has been suggested that Zn should be prescribed at the initial stages of inflammatory reactions,^34^ ^,^ ^35^ which is known to play a major role in tooth movement.^36^ Additionally, a number of studies have pointed out that Zn can only perform where its deficiency exists;^37^ thus, pretreatment supplementation administered in the present study might have reduced any chance of Zn inadequacy and, therefore, eliminated its possible impact on OTM. 

Despite the insignificant difference among our study groups, a decrease in OTM occurred from the first dose of Zn up to 20 ppm, but increased in Group 3 in which rats received 50 ppm. The decrease could be justified based on the proposed anti-resorptive effects of Zn, but the reason for the increase may not be as simple to explain. Cerovic et al^38^ also reported similar findings regarding alkaline phosphatase activity and *in vitro* bone nodule formation with increasing Zn concentrations, suggesting "biphasic effects" for this element in addition to other possible processes including cytotoxicity at higher doses.

Our histomorphometric findings showed no significant difference in osteoclast number among groups. This supports our clinical OTM data, but may seem contradictory to a number of former investigations demonstrating a down-regulating influence of Zn on osteoclastic resorptive potential and differentiation.^7^ ^,^ ^39^ ^,^ ^40^ Nevertheless, osteoclastic number may not necessarily have a positive association with osteoclast function.^41^ Holloway et al^42^ reported inhibition of bone resorption following Zn treatment in osteoblastic/osteoclastic co-culture, but found increased osteoclastic numbers. However, the study situation (*in vitro* versus *in vivo*), detection methods and Zn administration in their research were different from those used in the present investigation. 

The methods used in this study were selected based on previous research in this field;^7^ ^,^ ^43^ and, according to our findings, neither clinical nor histopathological changes were observed following Zn application in rats. Future studies using serum and/or urine analysis could help clarify the role of Zn in OTM and possibly confirm the conclusions of the current investigation. Additionally, further researches with immunohistochemical and molecular techniques are needed to understand the effect of Zn supplementation on bone remodeling during orthodontic treatment. If the present results are supported by future studies in animals and humans, the specific modifications in orthodontic treatment planning might not be necessary for patients receiving Zn as a dietary supplementation. 

Regarding the four locations of PDL width measured in our study, significant difference was found only in the distoapical aspect among groups. The reason for this difference is not clear; further studies on various histopathologic features of OTM following Zn treatment are suggested to help clarify the role of this important micronutrient in orthodontic treatment.

## CONCLUSIONS

According to the result obtained in the present study, systemic Zn supplementation up to 50 ppm does not affect OTM, neither bone nor root resorption in rats. Extrapolation of these findings to human subjects would require extensive research using more sophisticated techniques and drug concentrations.
