# ERRATUM: Effect of supplementary zinc on orthodontic tooth movement in a rat model

**DOI:** 10.1590/2177-6709.21.2.045-050.err

**Published:** 2016-10-17

In the article *Effect of supplementary zinc on orthodontic tooth movement in a rat model*, published at Dental Press J Orthod. 2016 Mar-Apr;21(2):45-50, where it is written:

Ahmad Akhoundi Mohammad Sadegh, Ghazanfari Rezvaneh, EtemadMoghadam Shahroo, Alaeddini Mojgan, Khorshidian Azam, Rabbani Shahram, Shamshiri Ahmad Reza, Momeni Nafiseh. 

Should be written:

Mohammad Sadegh Ahmad Akhoundi, Rezvaneh Ghazanfari, Shahroo Etemad-Moghadam, Mojgan Alaeddini, Azam Khorshidian, Shahram Rabbani, Ahmad Reza Shamshiri, Nafiseh Momeni.

